# In-vitro evaluation of the proliferative and osteogenic activity of atrophic non-union derived mesenchymal stem cells compared to autologous bone graft derived mesenchymal stem cells

**DOI:** 10.1007/s00068-026-03112-9

**Published:** 2026-02-16

**Authors:** Tim Niklas Bewersdorf, Jakob Hofmann, Laura Boehm, Sebastian Findeisen, Christian Schamberger, Thomas Lingner, Ulrike Sommer, Gerhard Schmidmaier, Tobias Grossner

**Affiliations:** 1 Faculty of Medicine, Heidelberg University, Heidelberg, 69120 Germany; 2 Heidelberg Trauma Research Group (HTRG), Clinic for Trauma and Reconstructive Surgery, Centre for Orthopaedics, Trauma Surgery and Spinal Cord Injury, Heidelberg University Hospital, Heidelberg, 69120 Germany; 3 Genevention GmbH, Rudolf-Wissell-Str. 28A, Göttingen, 37079 Germany; 4 Clinic for Trauma and Reconstructive Surgery, Centre for Orthopaedics, Trauma Surgery and Spinal Cord Injury, Heidelberg University Hospital, Schlierbacher Landstrasse 200a, Heidelberg, 69118 Germany

**Keywords:** Osteogenic activity, Proliferative activity, Bone-marrow mesenchymal stem cells, Atrophic non-union, Autologous bone graft, In-vitro

## Abstract

**Purpose:**

This study compares the proliferative and osteogenic activity of human bone marrow derived mesenchymal stem cells (MSCs) obtained from atrophic non-unions and from healthy autologous bone graft tissue (harvested from iliac crest or femoral canal) of the same patient in an in-vitro setting utilizing a matched control study design.

**Methods:**

MSCs underwent osteogenic differentiation over 3 weeks in-vitro (*n* = 6 donors; *n* = 36 samples/group) and the proliferative activity was accessed using DAPI-based immunofluorescence microscopy and WST-1 assay. All results regarding the osteogenic activity were normalized to 10^4^ cells to eliminate a proliferation bias. The late osteogenic activity was evaluated by radioactive ^99m^Technetium-hydroxydiphosphonate labelling of depleted hydroxyapatite, while early osteogenic markers (calcium concentration and alkaline phosphatase activity) were analysed in supernatants of cell culture media.

**Results:**

The early and late-stage osteogenic activity of atrophic non-union MSCs was significantly higher compared to healthy control graft MSCs on day 21 of osteogenic differentiation in-vitro, both in absolute numbers (early/late: *p* < 0.001) and after normalization to 10^4^ cells (early/late: *p* < 0.001). After lower proliferative activity during the first week, non-union MSC regained good proliferative activity during the second week resulting in comparable absolute cell counts to healthy control graft MSCs after three weeks.

**Conclusion:**

The results emphasize that the in-vitro osteogenic and proliferative activity of atrophic non-union MSCs is not impaired as clinically assumed but the osteogenic potential of atrophic non-union MSCs is in fact significantly higher compared to graft derived MSCs. This might be an important basic-science insight for the optimization of clinical non-union therapies.

## Introduction

The treatment of non-unions and the associated large size bone defects remains one of the greatest challenges in modern trauma and orthopaedic surgery [1–4]. Non-unions are categorized into atrophic and hypertrophic non-unions due to fundamental differences in their pathobiology and in their clinical treatment strategy [5–7]. While hypertrophic non-unions usually develop when the biomechanical stability is not given due to insufficient osteosynthesis, it is assumed that atrophic non-unions arise from poor local biological conditions or impaired vascularization [5–7]. Especially, the treatment of atrophic non-unions remains difficult as the poor local biological conditions result in an insufficient functional osteogenic activity [5, 7]. During the past decades several different therapies were developed and established to treat these conditions [3, 4, 8, 9]. For successful bony consolidation, it is crucial to understand the complex underlying pathobiology of non-unions and consequently treat the identified contributing factors [10, 11]. The Diamond Concept identified several factors, which are essential for bony regeneration and healing and therapies should address these factors to optimize clinical efficacy [10]. Osteogenic cells, in particular cells with the capability to produce extracellular bone matrix, like osteoblasts and osteogenic differentiated mesenchymal stem cells (MSCs) have been defined as one of the most important factors [10, 11].

For decades, harvesting autologous bone grafts (ABG) from the iliac crest followed by its transplantation to the non-union site has been the therapeutic gold standard [12, 13]. ABG contains osteoblasts, bone marrow-derived MSCs and growth factors supporting osseous regeneration [14–16]. The healing capacity of ABG is further improved utilizing a 2-step approach, known as the induced membrane technique, developed by Masquelet [17]. While ABG from the iliac crest is suitable for the treatment of small size bone defects (< 2–3 cm), disadvantages, like limited availability and extensive donor site morbidity, increase with the amount of harvested bone graft from this site [12, 13, 18]. Therefore, several authors recommend harvesting ABG from the medullary canal of long bones of the lower extremity, like femur and tibia, for larger bone defects [1, 3, 12, 19]. Since the clinical introduction of the Reamer-Irrigator-Aspirator (RIA) system in 2001 and its improvements in the following years it is now possible to obtain autologous bone grafts from long bones in 1.25 to 5-times larger amounts compared to the iliac crest with substantially lower donor site morbidity [3, 9, 20, 21]. Nevertheless, iliac crest is still the preferred harvesting site, as it contains higher numbers of MSCs in comparison to other harvesting sites [22–24].

Data from recent studies identified differences between ABG harvested via RIA and iliac crest both on a cellular and molecular level [1, 14–16]. While RIA cells showed predominantly elevated growth factor levels [16, 25] and higher osteocalcin levels [15] in comparison to iliac crest, other studies detected comparable cell viability [15], proliferation rates [24, 25] and overall osteogenic activity of cells harvested from both grafting sites [1]. Due to these ambiguous results, decisions about which harvesting site to use remain based on clinical considerations and surgeons’ preference.

Several clinical and patient derived risk factors of non-union formation have been identified and widely accepted. Hernigou et al. reported that bone marrow samples from NU sites and iliac crest of NU patients have in general lower levels of bone marrow progenitor cells compared to healthy controls most probably due to systemic factors, like smoking, alcohol abuse or chemotherapy [26]. Hence, they suggested that NU patients suffer from a generally impaired medullary stroma [26]. However, data regarding the functional osteogenic activity of non-union derived bone-marrow MSCs remains scarce. The functional osteogenic activity describes the ability of a tissue to produce extracellular bone matrix and depends on the cell amount and proliferative activity of osteogenic cells and osteogenic precursor cells like MSCs, and the osteogenic activity of these cells assessed by the amount of depleted extracellular bone matrix [27]. In particular, controversies remain regarding the proliferation rate [6, 7] and the osteogenic activity of cells in atrophic non-union tissue [6, 28–31]. Therefore, it is crucial to gain more knowledge about the individual proliferative and osteogenic activity of osteoblasts and MSCs derived from atrophic non-union tissue in comparison to MSCs derived from healthy bone graft tissue of the same patient to evaluate the functional osteogenic activity. This helps to get a better general understanding of the pathobiology and hereby ultimately contributing to an improvement of non-union therapy.

The aim of this study is to characterize and compare the functional osteogenic activity based on the proliferative and osteogenic activity of atrophic non-union and healthy graft (harvested via RIA or iliac crest) derived MSCs in a matched study design with further normalization of the result to 10^4^ cells to avoid a proliferation bias and to analyse the osteogenic potential of a single cell to get a better understanding of the cellular mechanisms for future improvement of clinical therapy.

## Materials and methods

### Harvesting, expansion, and osteogenic differentiation of MSC

#### Harvesting

**Fig. 1:**
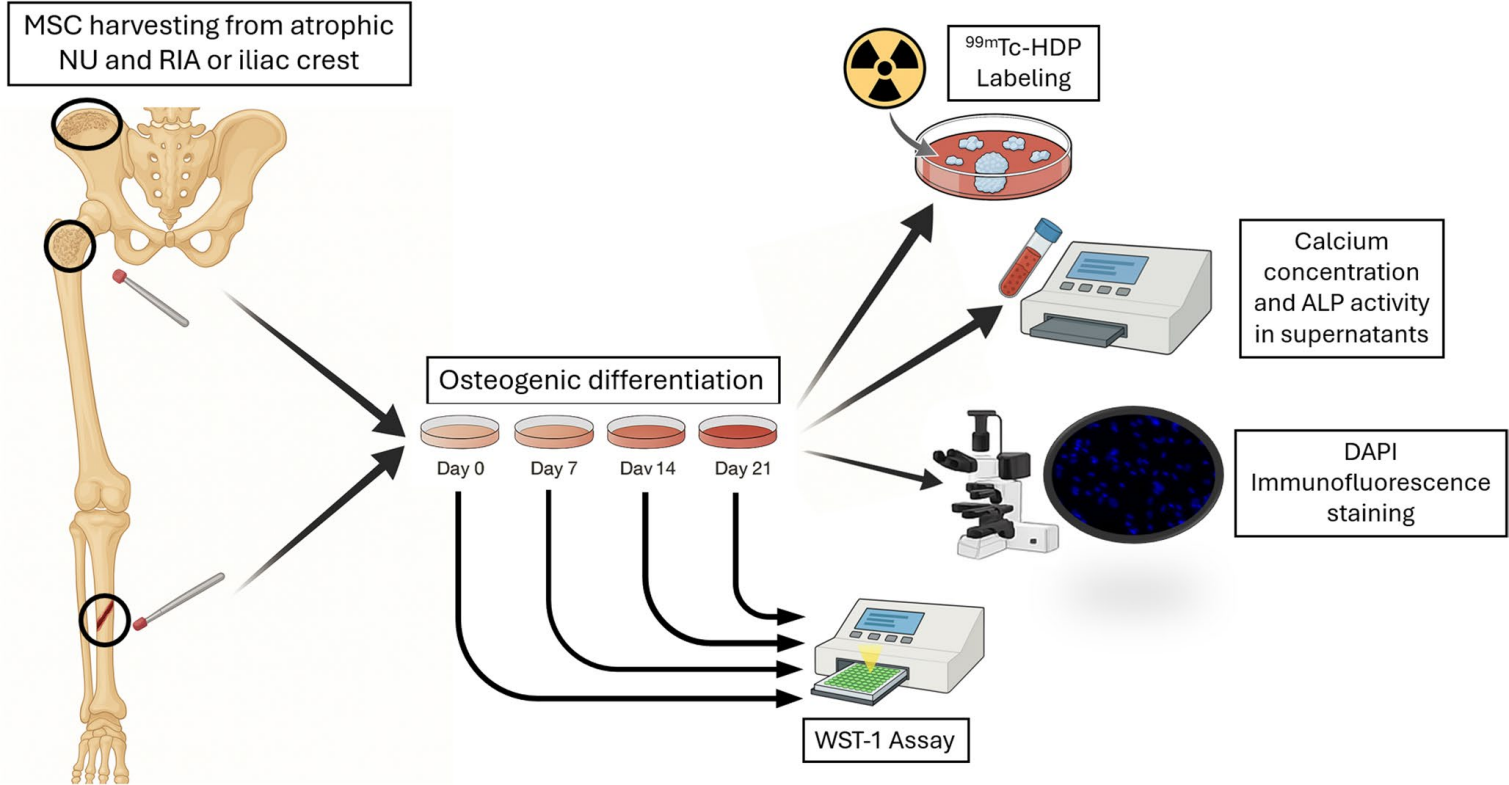
Experimental setup of the study. MSC: mesenchymal stem cells; NU: atrophic non-union; RIA: Reamer-Irrigator-Aspirator femur aspirate; ^99m^Tc-HDP: ^99m^Technetium-hydroxydiphosphonate; ALP: alkaline phosphatase

The experimental study protocol was approved by the Ethical Committee, Faculty of Medicine, Heidelberg University, Heidelberg, Germany (No. S-523/2023). All patients provided informed consent. Atrophic non-union and graft bone marrow samples from 6 patients (2 females, 4 males) were obtained during standard-of-care treatment following the 2-step Masquelet concept [17]. Patients suffering from diseases, which can influence the proliferative or osteogenic activity, like diabetes, osteoporosis, tumour diseases, or alcohol/drug abuse, were excluded per protocol. To eliminate other systemic influences like age, sex, or hormonal differences, we used graft derived MSCs from the same patient as an intrinsic control, as these systemic factors impact both NU-MSCs and GRA-MSCs. By referencing the NU-MSCs to the intrinsic graft derived control we annihilated inter-donor-varieties of the osteogenic potential and ensured that any detected difference in the functional osteogenic activity is based on differences between non-union and graft tissue and is independent of systemic factors. As it is well-known that graft tissue from different harvesting sites contains different amounts of MSC [22–24], it is important to normalize the cell count prior to any further cell culture experiment and according evaluation. Therefore, we initially isolated the MSC fraction from the various tissue samples of the iliac crest and RIA and then seeded an exact number of MSC in the cell culture dishes. This resolved the potential effects of various MSC densities in different tissues. The experimental setup is shown in Fig. 1, while detailed information about donors is provided in Table 1.

**Table 1:** Detailed information about donors: NU: atrophic non-union

Donor No.	Affected bone	Previous infection	sex	Graft origin	Months until NU diagnosis	Age	Patient related risk factors*
**1**	Radius	No	Male	Iliac crest	9	30	Smoker
**2**	Radius	No	Male	Iliac crest	9	29	None
**3**	Humerus	No	Male	Iliac crest	12	44	Smoker
**3**	Femur	Yes	Male	RIA	15	36	None
**4**	Femur	No	Female	RIA	12	49	None
**6**	Femur	No	Female	RIA	9	56	None

RIA: Reamer-Irrigator-Aspirator femur aspirate. *: risk factors: osteoporosis, severe obesity (body-mass-index ≥ 40), diabetes mellitus, positive smoking status, open fracture, alcohol/drug abuse, ASA grade ≥ 3

Non-union was diagnosed according to the definition of the Food and Drug Administration (FDA) when there was an inadequate osseous healing visible after 9 months [32]. The atrophic character was defined based on radiological, clinical, and histopathological parameters independently by two blinded consultants, experienced in non-union therapy. Atrophic non-union samples (*n* = 6) were collected by curettage of the bone at the non-union site after debridement of necrotic and scar tissue and 3–5 vital bone fragments of 5–10 mm size per patient were collected. Depending on the surgical procedure, healthy control graft cells were harvested from the femoral canal (*n* = 3) or iliac crest (*n* = 3) simultaneously to non-union cell collection. For harvesting the femoral canal, the RIA 2 system (DePuy Synthes, Westchester, Pennsylvania, USA) was used as recommended by the manufacturer. 3–5 ml of morselized bone marrow aspirate was collected from the femur, while 3 ml of iliac crest bone marrow derived ABG were taken with a surgical spoon after preparation of the iliac crest and opening the cortex with a chisel. Collected probes from iliac crest and RIA femur aspirate were identical to the ABG used in these patients. All samples were temporarily stored in 0.9% sdium chloride (NaCl) water with 5,000 IU of heparin and were transferred immediately to the cell culture laboratory.

Table 4 in the Appendix provides further details regarding the materials used in this study. Individual samples were washed twice with phosphate buffered saline (PBS, Gibco, Frankfurt, Germany). Subsequently, bone fragments were milled using a bone rongeur, seeded in polystyrene 100 mm-dishes (SARSTEDT, Nuembrecht, Germany) and cultured in high-glucose Dulbecco’s Modified Eagle’s Medium (DMEM, Gibco) containing 10% foetal bovine serum (FBS, Gibco) and 1% Penicillin/Streptomycin (Sigma, Schnelldorf, Germany). Flasks were coated with 0.5% gelatine (Sigma) in PBS for 30 min prior to use. After 48 h, all non-adherent cells were removed by washing with PBS to discard debris and cells of the hematopoietic lineage. Consequently according to the definition of Dominici et al. remaining plastic adherent cells were defined as MSCs [33].

#### Expansion and osteogenic differentiation

Passage 1 (P1) MSCs were seeded in gelatine coated 100 mm-dishes at a density of 1,666 cells/cm^2^ for expansion, which was performed using low-glucose DMEM (Gibco) with 10% FBS and 1% Penicillin/Streptomycin in a humidified 5% CO_2_ atmosphere at 37 °C. Medium was changed three times a week (every 2–3 days). After approximately 5 days cells reached a confluence rate of 80–90%. MSCs isolated from atrophic non-unions were labelled as NU, and healthy graft derived MSCs were labelled as GRA. Within this study, we used *n* = 6 donors with *n* = 6 biological replicates per donor for all experiments. Table 2 shows the defined groups for experiments.

**Table 2:** Study design and group definition: atrophic non-union derived mesenchymal stem cells (MSC) of every donor were used for NU and NU-Co. Graft derived MSC of every donor were used for GRA and GRA-Co. Sample sizes are multiplications of number of donors and number of biological replicates

Group	Osteogenic Differentiation	Harvesting site	Sample size (*n*)	Number of donors (*n*)	Number of biological replicates (*n*)
Atrophic non-union (NU)	Yes	Non-union - 2 x radius - 1 x humerus - 3 x femur	36	6	6
Graft (GRA): RIA femur aspirate and iliac crest derived MSC	Yes	Graft - 3 x RIA femur aspirate - 3 x iliac crest	36	6	6
Non-union Control (NU-Co)	No	Non-union - 2 x radius - 1 x humerus - 3 x femur	12	6	2
Graft control (GRA-Co)	No	Graft - 3 x RIA femur aspirate - 3 x iliac crest	12	6	2

Cells (P1) from every group were then seeded sixfold at a density of 1,000 cells/cm^2^ in 35 mm-dishes (SARSTEDT). Osteogenic differentiation was induced over 21 days by using DMEM low glucose with 10% FBS, 100 nM dexamethasone (Sigma), 50 µM L-ascorbic acid (Sigma) and 10 mM β-glycerol-phosphate (Sigma) with media change three times a week in a humidified 5% CO_2_ atmosphere at 37 °C. To confirm osteogenic differentiation, all osteogenic groups were compared to corresponding non-osteogenic control groups.

For every group, which underwent osteogenic differentiation, we established non-osteogenic control groups, which did not undergo osteogenic differentiation. Non-osteogenic NU control group (NU-Co) was formed of two non-union samples of every donor, while non-osteogenic graft control group (GRA-Co) was formed of two graft samples of every donor. Non-osteogenic control groups were incubated using DMEM low glucose (Gibco) with 10% FBS and 1% Penicillin/Streptomycin in a humidified 5% CO_2_ atmosphere at 37 °C without osteogenic differentiation. Methods were performed as displayed in Table 3.

**Table 3:** Methods overview

Analysed object	Method	Cell culture container	Tissue/supernatant	osteogenic marker timepoint (early or late)	Time-point [day]	Number of evaluated samples	Normed for 10^4^ cells
Cell viability and proliferation	WST-1 assay	96-well plate	Living cells during ongoing cell culture	-	7, 14, 21	36	No
Cell count	DAPI immunofluorescence	35 mm-dish	Fixated cells	-	21	NU/GRA: 36 Nu-Co/GRA-Co: 12	No
Calcium concentration	Photometric	35 mm-dish	Supernatant	Early	21	36	Yes
Alkaline phosphatase activity	Photometric	35 mm-dish	Supernatant	Early	21	36	Yes
Hydroxyapatite depletion	^99m^Tc-HDP labelling	35 mm-dish	Fixated cells	Late	21	NU/GRA: 36 Nu-Co/GRA-Co: 12	Yes

### Assessment of proliferative activity

#### Cell proliferation assay

To quantify cell proliferation and viability during osteogenic differentiation enzymatic conversion of tetrazolium salt was measured with the WST-1 cell proliferation kit (Merck, Darmstadt, Germany). Cell culture medium was replaced with fresh medium containing 1:10 volume of cell proliferation reagent WST-1. Cells were incubated for 20 min on day 0, 7, 14, and 21 of osteogenic differentiation. Enzymatic conversation to formazan was detected by colour change quantified at 450 nm using a microplate reader (FLUOstar Omega, BMG Labtech, Ortenberg, Germany). Reference wavelength was 650 nm. Results calculated by subtraction detected absorbance of reference wavelength from results of 450 nm measurements.

#### Absolute cell count determination

In this study, we used DAPI immunofluorescence microscopy for assessment of the absolute cell count after 21 days of osteogenic differentiation. Therefore, cells were first fixated with 70% ethanol (Sigma) at room temperature for 20 min and then stained with DAPI (1:1000 in PBS) (Thermo Fisher Scientific, Karlsruhe, Germany) for 5 min. Stained cells were counted under a Keyence BZ-X800 immunofluorescence microscope (Keyence, Neu-Isenburg, Germany) with a 40 x magnification. For each sample, 6 random vision fields were selected to form mean values, which were used for analysis. Cell counting was conducted via the “CellProfiler” software (Broad Institute, https://cellprofiler.org, version 4.2.1). Correct cell counting was manually checked. Mean values of each sample were calculated to norm other results to cell counts. Norming was performed to equalize cell count and proliferation dependent differences on the osteogenic potential. Absolute cell count was calculated by multiplication the area of the vision field to the entire cell culture dish.

### Assessment of osteogenic activity

####  Calcium concentration in supernatants

The early osteogenic activity was evaluated by determination of the calcium concentration in supernatants. Therefore, cell culture media were collected on day twenty-one and calcium concentration was measured photometrically using a Siemens Atellica CH Analyzer (Siemens Healthineers, München, Germany) with validated standards for analysing cell culture media. Atellica CH Calcium_2 (CA_2) kit (Siemens Healthineers, reference number: 11097644) was used to form coloured complexes of calcium ions with Arsenazo III, which was detected at 658 nm wavelength. Reference wavelength was 694 nm. The measured intensity of the detected complex is directly proportional to the calcium concentration in the supernatants.

#### Alkaline phosphatase activity in supernatants

To provide validated insights into the early osteogenic activity, we measured the well-established early osteogenic marker alkaline phosphatase (ALP) activity in supernatants collected on day twenty-one. ALP activity was quantified photometrically using the Atellica CH Alkaline Phosphatase (ALP_2c) kit (Siemens Healthineers, reference number: 11097600). ALP catalyses the transphosphorylation of p-nitrophenylphosphatase to p-nitrophenol, which was photometrically detected at 410 nm wavelength using a Siemens Atellica CH Analyzer (Siemens Healthineers) with validated standards for analysing cell culture media. Reference wavelength was 478 nm.

#### ^99m^Technetium-hydroxydiphosphonate labelling and analysis

Extracellular hydroxyapatite, depleted by osteogenic cells, is the most important marker for evaluation of the late osteogenic activity [34–37]. Therefore, high-sensitive quantitative radioactive ^99m^Technetium-hydroxydiphosphonate (^99m^Tc-HDP) labelling was chosen over Alizarin Red histological staining and subsequently performed. ^99m^Technetium-hydroxydiphosphonate binds selectively to newly synthesized hydroxyapatite and this uptake can be precisely quantified [34, 35]. As previously described, 5 MBq ^99m^Tc-HDP solved in 1 ml of 0.9% NaCl water were added to each dish [38, 39]. Incubation of the cells was performed for 15 min at room temperature, before the remaining solution was removed. Then, dishes were washed twice in PBS to remove the unbound radiotracer. Bounded ^99m^Tc-HDP was assessed by measuring gamma counts per dish during 5 s with a dose calibrator (Activimeter ISOMED 1010, Nuklearmedizintechnik Dresden, Dresden, Germany). Gamma counts were measured two times per probe, and the mean of each probe was used for analyses. ^99m^Tc-HDP uptake was measured in MBq of bounded ^99m^Technetium to the cell culture.

### Norming for 10^4^ cells and statistics

#### Norming for 10^4^ cells

The ability of a tissue to produce extracellular bone matrix depends on the number of osteogenic cells and the osteogenic activity of every single cell, as only enough osteogenic cells with a sufficient osteogenic activity can produce extracellular bone matrix in adequate amounts [27]. Consequently, the concentration and amount of osteogenic markers in cell culture dishes represent the functional osteogenic activity as it directly depends on the proliferative activity and the osteogenic activity of the seeded cells. Therefore, the determined values of the osteogenic markers used for assessment of the osteogenic activity represent the functional osteogenic activity as it is a result of all cells contained in the cell culture dish. Consequently, high proliferative activity and a subsequently higher number of cells can result in a skewed perception of individual osteogenic activity and therefore it is not possible by these results to draw a conclusion regarding the individual osteogenic activity of a single cell. To avoid a proliferation bias and to differ, whether an altered functional osteogenic activity represents altered osteogenic activity per cell or is a result of differing proliferative activity, we normalized results of all osteogenic markers to the level of 10^4^ cells based on the results of the DAPI cell count. For clear visualization of the data calcium concentration per 10^4^ cells is provided in µmol/10^4^ cells, ALP activity per 10^4^ cells in U/10^7^ cells, and ^99m^Tc-HDP uptake per 10^4^ cells in MBq/10^4^ cells.

#### Statistics

For statistical analysis of possible differences between NU and GRA a linear mixed model (LMM) for matched pair analysis was chosen and calculated. This model treats individuals as random effects within a model that has also fixed effects. We included a random intercept for the six individual donors to account for the dependence of samples originating from the same donor, while NU and GRA were defined as fixed effect. An unstructured covariance matrix was used for the random intercept to allow all variances and covariances to be estimated freely without imposing additional constraints. Parameters were estimated using restricted maximum likelihood (REML), and denominator degrees of freedom were approximated using the Kenward–Roger method to obtain small-sample-adjusted test statistics and confidence intervals. Mean values, standard deviations (SD), p-values for the fixed effect and intraclass correlation coefficients (ICC) for the random intercept are reported for LMM. Due to the small sample size in NU-Co and GRA-Co (*n* = 12 per group), we used the non-parametric Wilcoxon-signed-rank test for statistical analysis of NU and GRA compared to their corresponding NU-Co and GRA-Co (^99m^Tc-HDP uptake, DAPI cell count). Therefore, we calculated the mean values of all samples from one donor and used the mean values of each donor for statistical analysis. Medians, inter quartile ranges (IQR) and p-values are reported for non-parametric statistics. All measures of central tendencies of all results are also reported in Table 5 in the appendix. Statistical significance was set to *p* ≤ 0.05. SPSS Statistics Version 29 (IBM, Armonk, NY, USA) was used for statistical analysis and visualization. Graphics were created with SPSS Statistics Version 29, Microsoft Office Version 2506 (Redmont, WA, USA) and Microsoft Copilot.

## Results

### WST-1 cell proliferation assay

**Fig. 2:**
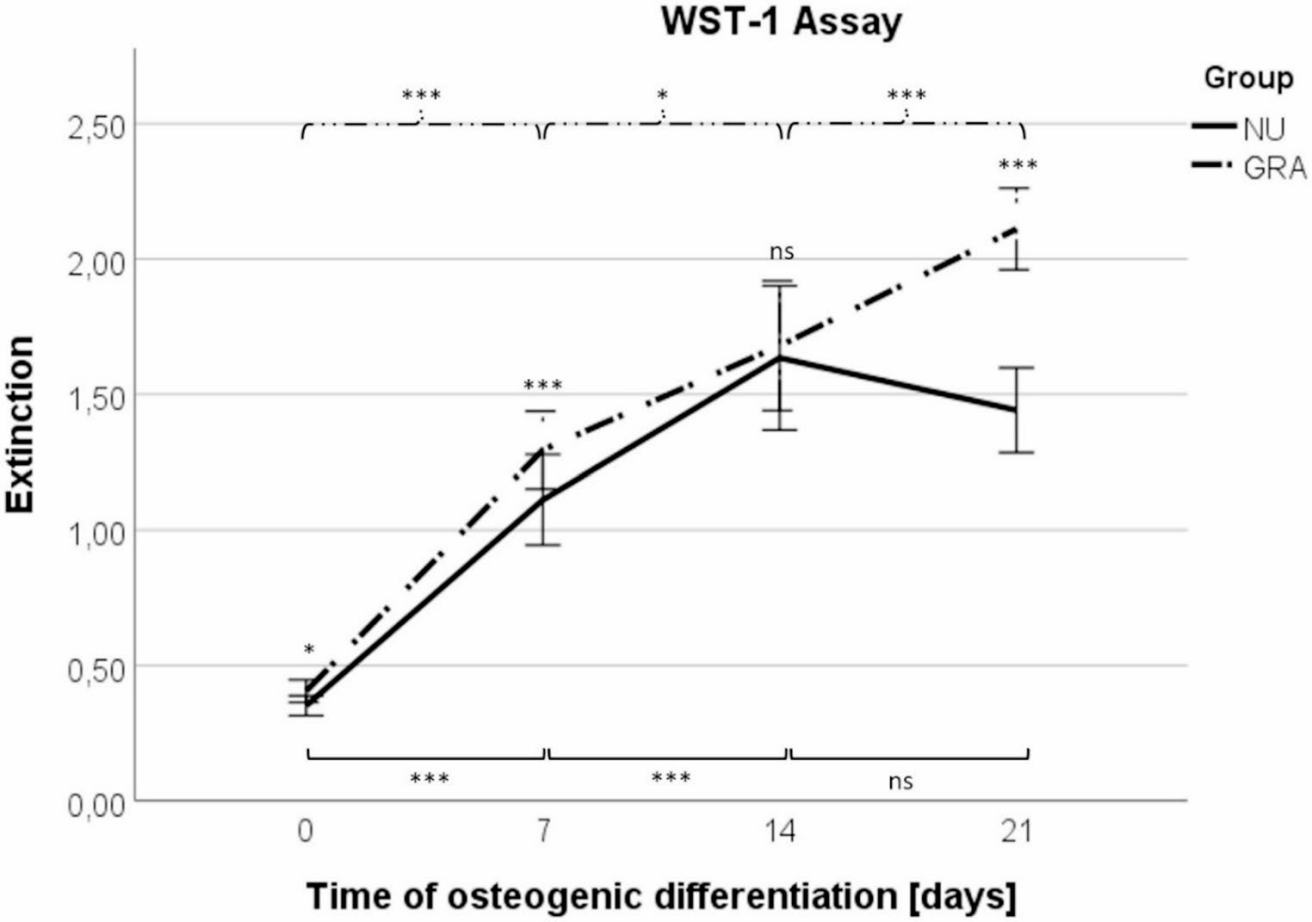
Line graph with error bars of mean values of WST-1 cell proliferation assay during ongoing osteogenic differentiation of 21 days in-vitro. The proliferation rates of both groups were measured on day 0, 7, 14, and 21 after 20 min incubation. Results were calculated by subtraction of reference wavelength. *n* = 6 donors, *n* = 6 samples per donor and timepoint, *n* = 1 technical replicate. Line: connects mean values of the groups. Whiskers: standard deviation within a specific group and timepoint. Significances are indicated by stars; stars without a bar show significance between NU and GRA within one timepoint; dashed bars under the stars indicate the compared GRA group of each significance. Solid bars above the stars indicate the compared NU group of each significance. NU: atrophic non-union derived mesenchymal stem cells. GRA: graft (RIA femur aspirate or iliac crest) derived mesenchymal stem cells. ns: not significant, *: *p* < 0.05, ***: *p* < 0.001

The proliferative activity of NU and GRA during ongoing osteogenic differentiation was assessed by WST-1 assays on day 0, 7, 14 and 21 and is displayed in Fig. 2. Within the NU group we detected a significant increase in the extinction over time during the first two weeks of osteogenic differentiation (day 0: mean: 0.35, SD: 0.108; day 7: mean: 1.11, SD: 0.493; day 14: mean: 1.63, SD: 0.787; day 21: mean: 1.44, SD: 0.463. Week 1 and week 2: *p* < 0.001), followed by a plateau during week three (*p* = 0.193). In contrast to NU results, we detected in GRA a significant increase of extinction over the whole period of osteogenic differentiation (day 0: mean: 0.41, SD: 0.110; day 7: mean: 1.37, SD: 0.446; day 14: mean: 1.66, SD: 0.667; day 21: mean: 2.13, SD: 0.411. Week 1: *p* < 0.001, week 2: *p* = 0.021, week 3: *p* < 0.001) without any plateau.

Comparisons of NU and GRA at the same time point revealed significantly higher extinction and therefore higher proliferation rate of GRA on day 0 (*p* = 0.016), day 7 (*p* < 0.001) and day 21 (*p* < 0.001). As increase in extinction in week 2 was higher in NU than in GRA, there was no significant difference in extinction on day 14 between both groups (*p* = 0.889) with only slightly higher extinction in GRA.

### DAPI cell count

**Fig. 3:**
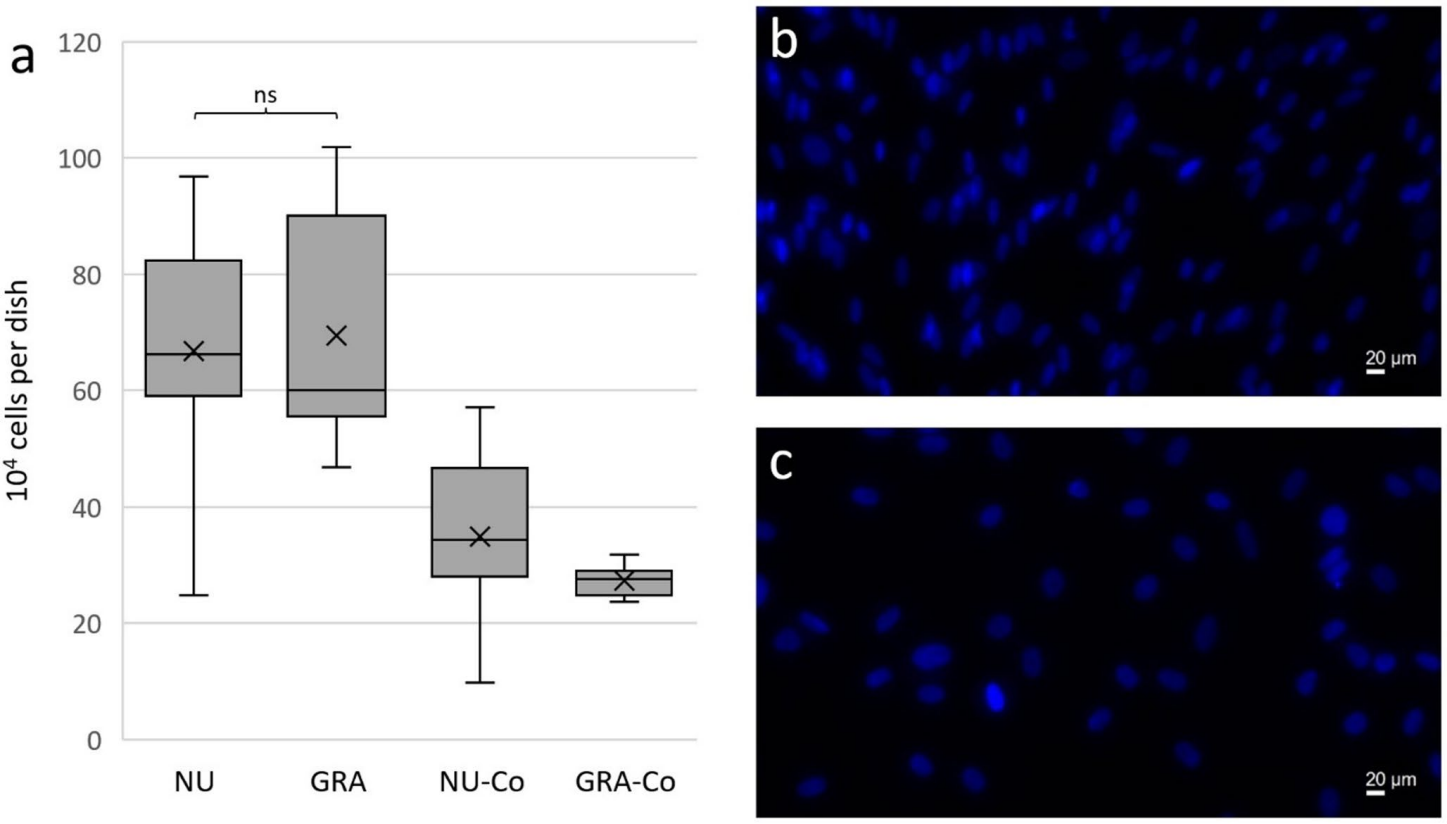
DAPI immunofluorescence microscopy cell count. **a**: box plot of cell counts per dish: mean cell amount per group assessed by DAPI immunofluorescence staining displayed as 10^4^ cells. Boxes: interquartile range, whiskers: 1.5 x interquartile range, horizontal line: median, x: mean. Significances are indicated by stars; bars under the stars indicate the compared group of each significance. NU: atrophic non-union derived mesenchymal stem cells. GRA: graft (RIA femur aspirate or iliac crest) derived mesenchymal stem cells. NU-Co: non-osteogenic differentiated control group for NU, which was formed out of samples of the same donor. GRA-Co: non-osteogenic differentiated control group for GRA, which was formed out of samples of the same donor. *n* = 6 donors, *n* = 6 samples per donor, *n* = 1 technical replicate, *n* = 6 vision fields per sample. ns: not significant. **b**: Example immunofluorescence image of GRA (iliac crest) on day 21. **c**: Example immunofluorescence image of corresponding GRA-Co on day 21

DAPI immunofluorescence staining of the dishes revealed no significant differences in total cell count between NU and GRA with slightly higher cell counts per dish in GRA (NU: mean: 668,476 cells/dish; SD: 204,239 cells/dish; median: 662,791 cells/dish; IQR: 233,681 cells/dish; GRA: mean: 695,014 cells/dish; SD: 190,968 cells/dish; median: 601,084 cells/dish; IQR: 344,372 cells/dish; LMM: *p* = 0.425; Fig. 3a). The ICC of all donors was 0.737. Both non-osteogenic control groups (NU-Co: median: 343,515 cells/dish; IQR: 187,002 cells/dish. GRA-Co: median: 276,668 cells/dish; IQR: 42,118 cells/dish) showed significantly lower cell counts than corresponding osteogenic groups (Wilcoxon-signed-rank test: NU vs. NU-Co: *p* = 0.028; GRA vs. GRA-Co: *p* = 0.043) without a significant difference between NU-Co and GRA-Co (Wilcoxon-signed-rank test: *p* = 0.345). Means and SDs, as well as medians and IQRs of all results are displayed in Table 5 in the appendix.

### Calcium concentration in supernatants

**Fig. 4:**
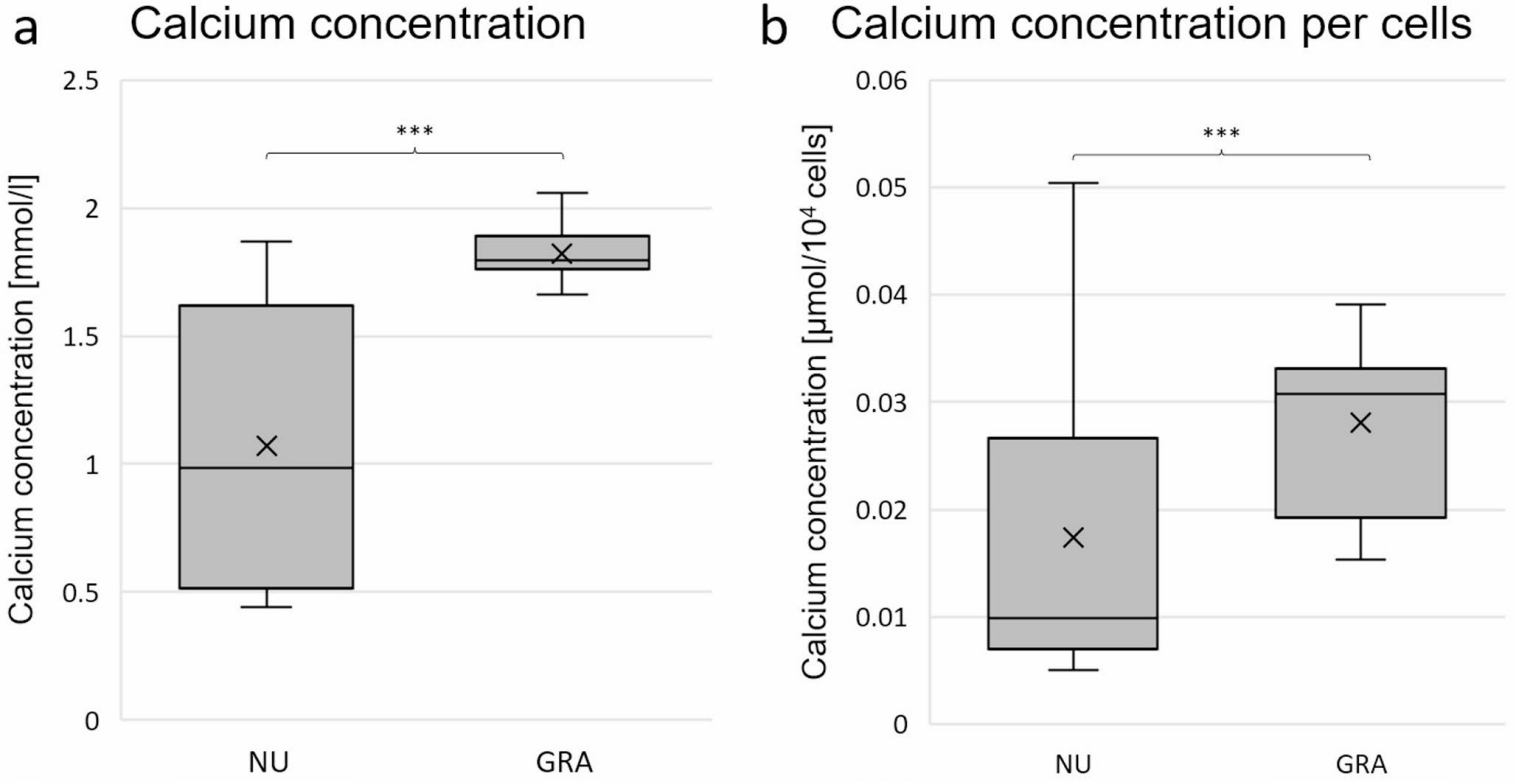
Calcium concentration in supernatants. **a**: box plot of calcium concentration in supernatants: mean calcium concentration in mmol/l in supernatants per group in-vitro. **b**: box plot of calcium concentration in $\:\frac{\mu\:mol}{{10}^{4}\:cells}$. Mean calcium concentrations in supernatants were normed for 10^4^ cells by dividing the results by average cell number of the same sample. *n* = 6 donors, *n* = 6 samples per donor, *n* = 1 technical replicate. Boxes: interquartile range, whiskers: 1.5x interquartile range, horizontal line: median, x: mean. Significances are indicated by stars. NU: atrophic non-union derived mesenchymal stem cells. GRA: graft (RIA femur aspirate or iliac crest) derived mesenchymal stem cells. ***: *p* < 0.001

Analysis of calcium concentrations in supernatants showed a significantly lower calcium concentration in NU (mean: 1.070 mmol/l; SD: 0.555 mmol/l) compared to GRA (mean: 1.820 mmol/l; SD: 0.098 mmol/l; LMM: *p* < 0.001; Fig. 4a). The calculated ICC was 0.514. To evaluate the osteogenic activity of a single cell and to eliminate a potential proliferation bias, all osteogenic markers were normed for 10^4^ cells based on the absolute DAPI cell count for each group. Calcium concentration per cell revealed comparable results to absolute calcium concentrations, as shown in Fig. 4b. Differences in calcium concentration per 10^4^ cells were significant with *p* < 0.001 in NU (mean: 0.0174 µmol/10^4^ cells; SD: 0.0135 µmol/10^4^ cells) compared to GRA (mean: 0.0281 µmol/10^4^ cells; SD: 0.0072 µmol/10^4^ cells) with an ICC of 0.622. Means and SDs, as well as medians and IQRs of all results are displayed in Table 5 in the appendix.

### Alkaline phosphatase activity in supernatant

**Fig. 5:**
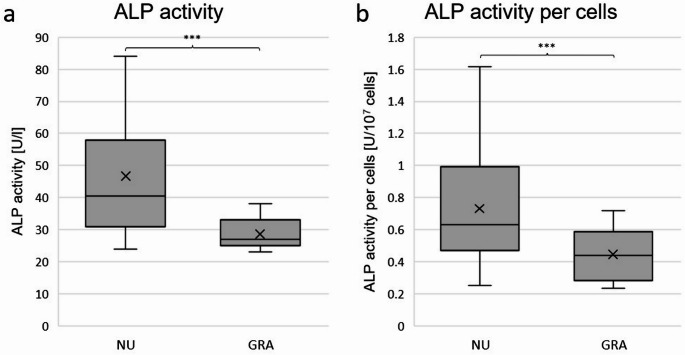
ALP activity in supernatants. **a**: box plot of mean ALP activity detected in supernatants: mean ALP activity measured in U/l in supernatants per group in-vitro. **b**: box plot of ALP activity displayed in $\:\frac{U}{{10}^{7}\:cells}$. Mean ALP activity was normed for 10^4^ cells by dividing the results by average cell number of the same sample *n* = 6 donors, *n* = 6 samples per donor, *n* = 1 technical replicate. Boxes: interquartile range, whiskers: 1.5x interquartile range, horizontal line: median, x: mean. Significances are indicated by stars. NU: atrophic non-union derived mesenchymal stem cells. GRA: graft (RIA femur aspirate or iliac crest) derived mesenchymal stem cells. ***: *p* < 0.001

A significantly higher mean ALP activity was measured in NU (mean: 46.694 U/l; SD: 17.674 U/l) compared to GRA (mean: 28.694 U/l; SD: 4.732 U/l; LMM: *p* < 0.001) as displayed in Fig. 5a. ICC showed a strong correlation of samples within the same donor, as ICC was 0.523. After norming the mean ALP activity to 10^4^ cells, ALP activity per cell was still significantly higher in NU (mean: 0.7315 U/10^7^ cells; SD: 0.3646 U/10^7^ cells) than in GRA (mean: 0.4468 U/10^7^ cells; SD: 0.1473 U/10^7^ cells; LMM: *p* < 0.001; Fig. 5b). The calculated ICC was 0.596. Means and SDs, as well as medians and IQRs of all results are displayed in Table 5 in the appendix.

### ^99m^Tc-HDP labelling

**Fig. 6:**
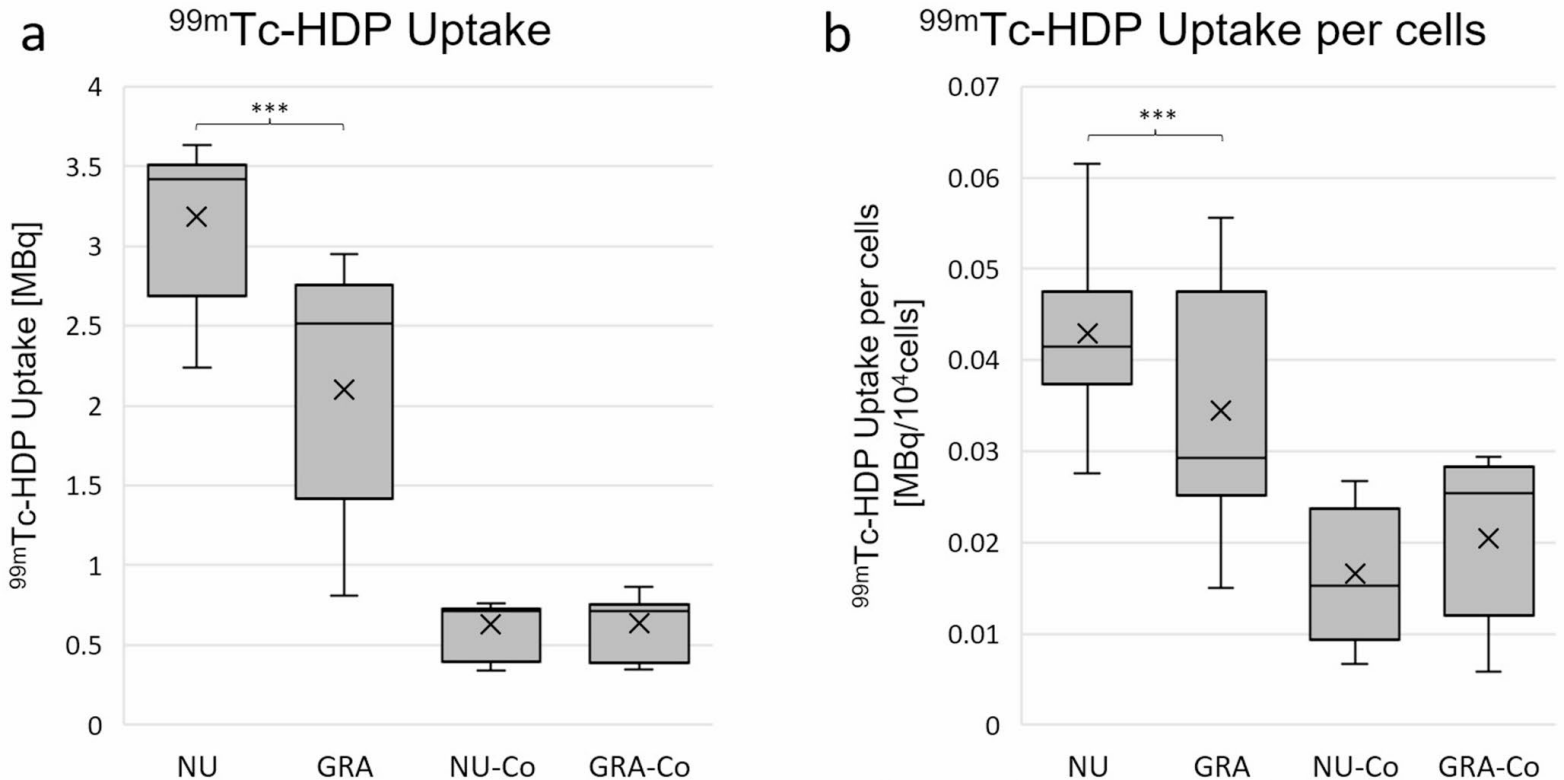
^99m^Technetium-hydroxydiphosphonate (^99m^Tc-HDP) uptake. **a**: box plot of mean ^99m^Tc-HDP uptake in MBq: mean ^99m^Tc-HDP uptake per group in-vitro. **b**: box plot of ^99m^Tc-HDP uptake in MBq per 10^4^ cells. Mean ^99m^Tc-HDP uptake per group was normed for 10^4^ cells by dividing the results by average cell number of the same sample. *n* = 6 donors, NU/GRA: *n* = 6 samples per donor, NU-Co/GRA-Co: *n* = 2 samples per donor, *n* = 2 technical replicates (gamma count measurements). Boxes: interquartile range, whiskers: 1.5x interquartile range, horizontal line: median, x: mean. Significances are indicated by stars; bars under the stars indicate the compared group of each significance. NU: atrophic non-union derived mesenchymal stem cells. GRA: graft (RIA femur aspirate or iliac crest) derived mesenchymal stem cells. NU-Co: non-osteogenic differentiated control group for NU, which was formed out of samples from the same donor. GRA-Co: non-osteogenic differentiated control group for GRA, which was formed out of samples from the same donor. ns: not significant, ***: *p* < 0.001

Absolute ^99m^Tc-HDP uptake was more than three-times higher in GRA (mean: 2.102 MBq; median: 2.512 MBq; SD: 0.751 MBq; IQR: 1.340 MBq) than in GRA-Co (median: 0.714 MBq; IQR: 0.365 MBq) and more than five-times higher in NU (mean: 3.184 MBq; median: 3.417 MBq; SD: 0.463 MBq; IQR: 0.820 MBq) compared to NU-Co (median: 0.725 MBq; IQR: 0.332 MBq), which reflects a solid and significant hydroxyapatite depletion and therefore osteogenic differentiation due to the presence of osteogenic differentiation medium in NU and GRA (Fig. 6a). P-values of the Wilcoxon-signed-rank test were *p* = 0.028 for NU compared to NU-Co and *p* = 0.046 for GRA compared to GRA-Co. Moreover, we detected a higher absolute ^99m^Tc-HDP uptake in NU than in GRA (LMM: *p* < 0.001). The calculated ICC showed a moderate correlation of samples from the same donor (ICC: 0.373). As the mean ^99m^Tc-HDP uptake in NU-Co and GRA-Co was nearly the same, there were no significant differences between these groups (Wilcoxon-signed-rank test: *p* = 0.753).

For further analyses, we calculated the ^99m^Tc-HDP uptake per 10^4^ cells quotient (Fig. 6b). Similar to the total results, ^99m^Tc-HDP uptake normed for 10^4^ cells revealed significantly higher hydroxyapatite depletion in NU (mean: 0.0429 MBq/10^4^ cells; median: 0.0415 MBq/10^4^ cells; SD: 0.0134 MBq/10^4^ cells; IQR: 0.0101 MBq/10^4^ cells) in comparison to GRA (mean: 0.0345 MBq/10^4^ cells; median: 0.0293 MBq/10^4^ cells; SD: 0.0135 MBq/10^4^ cells; IQR: 0.0223 MBq/10^4^ cells; LMM: *p* < 0.001). Furthermore, NU showed a significantly higher ^99m^Tc-HDP uptake in comparison to NU-Co (Wilcoxon-signed-rank test: *p* = 0.046), while GRA and GRA-Co differed not significantly (Wilcoxon-signed-rank test: *p* = 0.173), most probably due to the small sample size of GRA-Co. Comparison of NU-Co (mean: 0.0166 MBq/10^4^ cells; median: 0.0153 MBq/10^4^ cells; SD: 0.0302 MBq/10^4^ cells; IQR: 0.0174 MBq/10^4^ cells) and GRA-Co (mean: 0.0205 MBq/10^4^ cells; median: 0.0253 MBq/10^4^ cells; SD: 0.0222 MBq/10^4^ cells; IQR: 0.0164 MBq/10^4^ cells) revealed no differences in ^99m^Tc-HDP uptake per cell (Wilcoxon-signed-rank test: *p* = 0.600). Means and SDs, as well as medians and IQRs of all results are displayed in Table 5 in the appendix.

## Discussion

Formation of an extracellular mineralized bone matrix, which ensures robust biomechanical stability, is the primary endpoint of any successful bone regeneration and non-union healing. Therefore, a sufficient functional osteogenic activity, which describes an adequate amount of cells with a solid proliferative and osteogenic activity, is crucial for this process [10, 11, 27]. The transplantation of biologically active bone grafts derived from autologous donor sites, like iliac crest and RIA femur aspirate, can provide sufficient amounts of such cells at the non-union site. Thus, transplantation of ABG remains as the most common technique for the treatment of large size bone defects and is considered to be the gold standard [3, 12, 13, 18, 40]. Preexisting studies showed that iliac crest and RIA derived osseous cells maintain a good proliferative and osteogenic activity [1, 15, 16], whereas studies investigating the functional osteogenic, osteogenic or proliferative activity of non-union derived MSCs provided controversial results and it remains unclear whether there are significant differences in the functional osteogenic, osteogenic or proliferative activity of MSCs derived from non-union compared to healthy bone graft within the same patient. Furthermore, hypertrophic and atrophic non-union were not distinguished in multiple studies and so differences of the osteogenic and proliferative activity of hypertrophic and atrophic non-union are not fully understood.

Histopathological studies demonstrated that atrophic non-union tissue consists of a mix of fibrous and connective tissue with mostly fibroblast-like cells in the vicinity of areas of necrotic bone and low cell density [6, 30, 31, 41, 42] as well as areas with high bone turnover and new bone formation [28, 41]. Nevertheless, findings from our study show that atrophic non-union samples of all donors possessed adequate numbers of MSC. This is in line with results from Cuthbert et al., who found similar numbers of MSCs in atrophic non-union tissue in comparison to surrounding healthy control tissue [31], and Ismail et al., who reported comparable MSC numbers in atrophic non-union and iliac crest tissue [7]. Despite this, we detected a lower extinction in WST-1 assay in NU compared to GRA on day 0 and 7 of osteogenic differentiation and consequently the early proliferative activity of NU-MSC was significantly lower. This is in line with a study conducted by El-Jawhari, who found a lower proliferative potential of uncultured non-union MSCs and lower response levels to growth factors compared to uncultured healthy bone marrow MSCs [43]. During the second week of osteogenic differentiation, we detected a convergence of the proliferative activity of NU and GRA, which resulted in nearly identical extinction in WST-1 assay on day 14 and is consistent with El-Jawhari et al. as they reported a narrowing of the proliferative activity of non-union and healthy bone marrow MSCs during ongoing cell culture [43]. Comparable proliferative activity during ongoing cell culture can be caused by restoration of adequate response to growth factors [43]. According to other studies, high concentrations of pro-proliferative markers, like vascular endothelial growth factor (VEGF) and Cyclin D1, were found in non-union tissue [31, 41, 44]. Furthermore, induced membrane tissue contains adequate numbers of MSC and expresses after several months bone tissue characteristics and bone matrix markers, like fibronectin, collagen type I, ALP and osteocalcin, but nevertheless failed to mineralize [45]. Consequently, samples isolated from non-union tissue contain comparable numbers of MSCs, which are under the high influence of pro-proliferative factors resulting in comparable cell counts assessed by DAPI staining after 21 days of osteogenic differentiation. The differences observed during the early timepoint regarding the osteogenic and proliferative properties could indicate that the local microenvironment at the non-union site promotes a potential hibernation state of the bone cells and therefore it is possible that the microenvironment rather than the cellular properties contributes to the impaired biology in atrophic non-unions.

Although ABG derived MSCs are harvested from healthy bone with physiological proliferative activity, the relatively rough harvesting procedure can affect the proliferative activity of these cells in-vitro. In detail, we detected in our study a slightly lower proliferative activity in RIA probes in comparison to iliac crest samples on day 7 (Appendix Fig. 7). This slight difference in proliferative activity was only visible during the first week of in-vitro osteogenic differentiation and the proliferative activities of iliac crest and RIA cells according to WST-1 assay did not differ on day 14 and 21. This resulted in no significant differences in absolute cell count on day 21 according to DAPI immunofluorescence staining. Comparing these results with previous studies, which focused on differences between RIA and iliac crest derived MSCs, viability of cells from these tissues was determined equal, with viability rates ranging from 95% to 99% [15, 46]. Nevertheless, El-Jawhari et al. found that primary RIA waste derived cells, especially RIA derived CD45^low^CD271^high^ cells, which are a surrogate for MSCs in freshly collected samples, have higher stress levels, indicated by higher reactive oxygen species (ROS) levels [46]. Also, they have a higher death susceptibility than cells collected from iliac crest [46]. Furthermore iliac crest and RIA waste derived cells showed higher ROS levels and a lower viability after an in-vitro RIA harvesting simulation [46]. On the other side, after two weeks of osteogenic differentiation in-vitro, the RIA waste and iliac crest derived MSCs exhibited comparable osteogenic and proliferative activities [46]. Another contributing factor to initially lower proliferative activity in RIA cells could be the harvesting site itself, as Davies et al. found significantly lower mononuclear cell counts in femur samples, as well as lower colony forming unit fibroblast (CFU-F) in cell cultures harvested from the femur compared to iliac crest derived cells after 14 days in-vitro [24]. In line with our study, they also detected no differences in proliferative activity and viability in later stages of in-vitro cultivation [24]. Consequently, the temporarily lower extinction in the samples of RIA derived MSCs detected by WST-1 assay on day 7 indicates that there is a transient reduced proliferative activity due to the harvesting procedure and the generally lower MSC density in the medullary canal of the femur compared to the iliac crest. Within two weeks of osteogenic differentiation the extinction and therefore the proliferative activity adapted properly. After restoration of the proliferative activity of RIA MSCs, cell counts of GRA were comparable to NU and the proliferative activity after 21 days was significantly higher.

As we detected the highest difference in proliferative activity of NU and GRA on day 21, it will be subject of further studies with extended time of in-vitro cultivation to find out whether proliferative activity in graft derived MSCs in the late phase of osteogenic differentiation remain high with consecutive higher absolute cell counts after more than 21 days or whether higher proliferative activity after 21 days is just an adaptive reaction to the transient harvesting-induced reduced proliferative activity and proliferative activity of non-union MSCs and graft MSCs will be similar after culturing cells for more than three weeks.

It is well-known that MSCs have two major roles in the human body, as they are essential for regeneration of mesenchymal tissue: proliferation to provide a sufficient cell amount and differentiation to fulfil the intended function of regenerated tissue [47, 48]. To preserve the ability of multilineage differentiation, MSCs proliferate first before they differentiate [47–50]. Therefore, flattening proliferative activity can indicate this switch from primarily proliferation to primarily differentiation and forming of extracellular bone matrix. The amount of extracellular hydroxyapatite is one of the most important markers to evaluate the late-stage osteogenic activity [34–37]. Extracellular hydroxyapatite depletion was accessed by quantitative measurement of ^99m^Technetium bounded to hydroxydiphosphonate, which directly binds selectively to newly formed hydroxyapatite [34]. Thus, this uptake can be precisely quantified using an activimeter to measure the amount of bound radioactive tracer [34]. The early osteogenic activity can be indirectly evaluated by quantitative assessment of osteogenic proteins, like ALP, or concentration analysis of the bone substrate calcium as calcium concentration in supernatants acts directly inverse to the osteogenic activity [36–38, 51, 52].

A significantly higher early osteogenic activity was detected in NU in comparison to GRA, as we found lower calcium concentrations and higher ALP activity in NU than in GRA. Moreover, a significantly higher hydroxyapatite depletion was measurable and consequently the late-stage osteogenic activity in NU probes was also higher in comparison to GRA. Although the osteogenic activity of NU-MSCs was clearly higher, we observed also a solid osteogenic activity of GRA-MSCs. This substantial osteogenic activity of NU and GRA in-vitro is in line with several other studies, in which successful osteogenic differentiation of MSCs from iliac crest [1, 6, 14, 15], RIA [1, 14, 15] and non-union [6, 28, 31, 42, 46] was proven. Furthermore, Gindraux et al. detected adequate numbers of MSCs with good osteogenic differentiation capability in the induced membrane tissue six months after the first Masquelet surgery [45]. While graft derived MSC were harvested from healthy bones with physiologically low bone turnover, and lower concentrations of pro-proliferative and pro-osteogenic factors [53, 54], several studies detected high concentrations of pro-proliferative and pro-osteogenic markers in non-union tissue [31, 41, 44]. Han et al. found areas in non-union samples with high expression of bone morphogenic protein 2 (BMP-2) and decorin, which promote formation of bone matrix and calcium deposition [44] and Schira et al. detected an up-regulation of WNT5A, which causes an up-regulation of the pro-osteogenic genes runt-related transcription factor 2 (RUNX2), osterix and ALP [41, 55]. This high concentration of pro-osteogenic factors can enhance the osteogenic activity of samples isolated from non-union tissue.

The functional osteogenic activity measured by the absolute amount of osteogenic markers depends on their production by every individual osteogenic cell and the number of osteogenic cells. To differentiate whether a higher functional osteogenic activity is a consequence of a higher osteogenic activity or a previous higher proliferative activity and subsequently higher number of osteogenic cells we performed a normalization for 10^4^ cells of all osteogenic markers. After normalization for 10^4^ cells both early (ALP activity and calcium concentration) and late osteogenic markers (hydroxyapatite depletion) continued to indicate a significantly higher osteogenic activity per cell in NU samples in comparison to GRA. Although absolute cell count in GRA was only slightly higher, this illustrates clearly the high osteogenic activity of atrophic non-union derived MSCs in this study, even after correcting for cell count to avoid a potential proliferation bias. This finding is remarkable, as there is no other publication which showed such high osteogenic activity of atrophic non-union derived MSCs in comparison to healthy controls. The reason for a lack of comparable data lies in the heterogeneity of existing studies and their study design. As other studies not solely focused on atrophic non-unions, and used different harvesting procedures, differentiation protocols and methods to assess the osteogenic and proliferative activity a cross-study comparison is difficult. Moreover, different MSC densities of various graft tissues result in alterations of absolute amounts of osteogenic markers. To minimize effects of differing MSC densities of iliac crest, RIA and non-union tissue on the functional osteogenic activity in this study isolated MSCs were seeded with an exact number of cells to ensure that the initial cell amount at the beginning of osteogenic differentiation did not differ between every sample. Due to this procedure no significant differences in the proliferative and osteogenic activity of iliac crest and RIA femur aspirate derived cells were detected in this study.

The calculated ICCs in this study revealed that there was a moderate to strong intra-donor correlation of all samples. The strongest correlation was detected for cell count (ICC: 0.737), followed by early osteogenic activity (ICC: 0.514–0.622) and late osteogenic activity (ICC: 0.373–0.421). This detected intra-donor correlation underlines that the functional osteogenic activity depends not only on the harvesting site, but also on the individual donor. Therefore, our study is in line with several other studies, as they also detected a inter-donor-variety of the proliferative and osteogenic activity [26, 28, 29]. We took this inter-donor-variety and the intra-donor-correlation into account by using the LMM for statistical analysis and revealed a significantly higher early and late osteogenic activity of atrophic non-union MSCs compared to healthy control graft MSCs both in absolute numbers and after normalization.

Most studies compared non-union MSCs with bone marrow MSCs of a different and healthy donor, but not with an intrinsic control where both specimens were obtained from the same patient and therefore inter-donor-varieties could influence the results [26, 28, 29]. Only a few studies have actually investigated cells derived from the same donor [6, 7, 41]. Although these studies indicate a good proliferative and osteogenic potential, the results of these studies were limited in no normalization to cell count and therefore a potential proliferation bias cannot be ruled out [6, 7, 41]. Thus, our study builds on that, and our results indicate that atrophic non-union MSC possesses a superior osteogenic activity over graft MSC, although proliferative activity was lower, but without any significant effects on total cell counts.

We analysed the proliferative and osteogenic activity over 21 days. Although standard osteogenic differentiation of MSCs over 21 days is quite common in in-vitro research, it is barely comparable to non-union healing in human with a mean consolidation time of up to 16 months in large size bone defects [56]. To our knowledge, as of today no proper in-vitro model exists to reproduce the complex situation in-vivo. For future investigation in this field, in-vitro studies over a longer period in a 3D-setting are under preparation to better reflect the in-vivo conditions.

Non-unions in general are a rare disease, and due to fundamental differences in their pathobiology a further subdivision into hypertrophic and atrophic, it is necessary to extract any valuable scientific data. As atrophic non-unions arise from poor local biological conditions or impaired vascularization [5–7], aim of this study was the assessment of the functional osteogenic activity of atrophic non-unions and the corresponding autologous bone grafts exclusively. Therefore, we present in this publication a structured assessment of the osteobiology of 6 human donors, representative for the pathology of atrophic non-unions. It is very challenging, even for highly specialized centres, to gather a large patient collective and their biological samples for an in-vitro assessment. That is probably the main reason why, to our knowledge, solely Vallim et al. utilized a larger sample size with a matched study design, but they did not normalized for cell count [6]. The combination of very low incidence and a high heterogeneity of existing studies limit comparability to only a few studies and highlights the need for further research. Our data should serve in the future as the basis for a multi-centre study setting, which could support a much higher overall sample size, to confirm our current observations. Due to inter-donor-varieties and intra-donor correlation regarding the osteogenic differentiation capacity of MSCs, matching of non-union and graft samples from the same patient, as we did in this matched-pair analysis, is essential for any insightful future research in that field [57–59].

This study shows that MSCs derived from atrophic non-unions possess a higher functional osteogenic activity and higher osteogenic activity per cell compared to graft derived MSCs in-vitro. If these findings are confirmed in further confirmatory in-vivo studies, our research strengthens the view that NU tissue has an excellent functional osteogenic activity. Proper local activation of proliferation and osteogenic differentiation of these MSCs by optimization of the local microenvironment can therefore become essential for healing of atrophic non-unions.

In conclusion, this study revealed significantly higher early and late osteogenic activity of atrophic non-union MSCs compared to healthy control graft MSCs on day 21 of osteogenic differentiation in-vitro both in absolute numbers and after normalization. Although the proliferative activity was lower during the first week of osteogenic differentiation, non-union MSC regained good proliferative activity during the second week with a subsequent decrease of proliferative activity due to ongoing osteogenic differentiation, ending up in similar cell counts after three weeks of osteogenic differentiation. Taken together this data indicates that atrophic non-union tissue contains sufficient numbers of MSCs resulting in an adequate functional osteogenic activity. Therefore, termini like “hypotrophic” or “altered osteobiology” describe these non-union entities better than atrophic. Ultimately, osteogenic differentiation of the locally existing MSCs could play a key role in future non-union therapy, especially if the defect is small enough to treat it without ABG transplantation or graft expanders.

## Figures and Tables

**Fig. 7:**
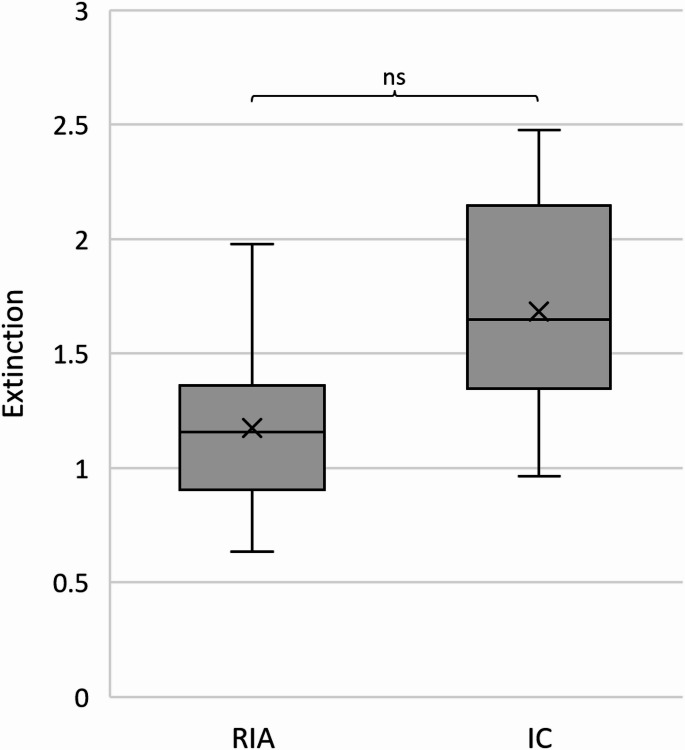
Box plot of WST-1 Assay – Day 7. WST-1 cell proliferation assay of RIA and IC on day 7: mean absorbance values per group after 20 min incubation in-vitro. Results were calculated by subtraction of absorbance of reference wavelength. *n* = 3 donors per group, *n* = 6 samples per donor, *n* = 1 technical replicate. Boxes: interquartile range, whiskers: 1.5x interquartile range, horizontal line: median, x: mean. Significances are indicated by stars; bar under the stars indicate the compared group of each significance. RIA: femur aspirate derived mesenchymal stem cells harvested by Reamer-Irrigator-Aspirator technique. IC: Iliac crest derived mesenchymal stem cells. ns: not significant

## Data Availability

The data that support the findings of this study are available from the corresponding author upon reasonable request.

## Appendix

**Table 4:** Reagents, manufacturer, and reagent Dilution used in this study

Reagent	Manufacturer	Dilution
Atellica CH Alkaline Phosphatase (ALP_2c) kit	Siemens Healthineers	-
Atellica CH Calcium_2 (CA_2) kit	Siemens Healthineers	-
β-glycerol-phosphate	Sigma, Schnelldorf, Germany	10 mM in DMEM low glucose
DAPI	Thermo Fisher Scientific, Karlsruhe, Germany	1:1000 in PBS
Dexamethasone	Sigma, Schnelldorf, Germany	100 nM in DMEM low glucose
DMEM (Dulbecco’s Modified Eagle’s Medium)	Gibco, Frankfurt, Germany	-
DMEM low glucose	Gibco, Frankfurt, Germany	-
Ethanol (70%)	Sigma, Schnelldorf, Germany	-
FBS (foetal bovine serum)	Gibco, Frankfurt, Germany	10% in DMEM and DMEM low glucose
Gelatine	Sigma	0.5% in PBS
Heparin	LEO Pharma, Neu-Isenburg, Germany	1000 IE/ml Sodium chloride 0.9% water
L-ascorbic acid	Sigma, Schnelldorf, Germany	50 µM in DMEM low glucose
PBS (phosphate buffered saline)	Gibco, Frankfurt, Germany	-
Penicillin/streptomycin	Sigma, Schnelldorf, Germany	1% in DMEM and DMEM low glucose
Sodium chloride 0.9% water	Fresenius Kabi, Bad Homburg, Germany	-
^99m^Technetium-hydroxydiphosphonate	Department of Nuclear Medicine, University Hospital Heidelberg, Heidelberg, Germany	5 MBq/ml of sodium chloride 0.9% water
WST-1 cell proliferation kit	Merck, Darmstadt, Germany	1:10 in DMEM

**Table 5:** Results overview

Method	Group	Mean	SD	Median	IQR
DAPI immuno-fluorescence [cells/dish]	NU	668,476	204,239	662,791	233,681
	GRA	695,014	190,968	601,084	344,372
	NU-Co	348,849	153,000	343,515	187,002
	GRA-Co	273,562	27,286	276,668	42,118
Calcium concentration [mmol/l]	NU	1.070	0.555	0.985	1.108
	GRA	1.820	0.098	1.795	0.013
Calcium concentration per cells [µmol/10^4^ cells]	NU	0.0174	0.0135	0.0099	0.0196
	GRA	0.0281	0.0072	0.0307	0.0139
ALP activity [U/l]	NU	46.694	17.674	40.500	27.000
	GRA	28.694	4.732	27.000	8.000
ALP activity per cells [U/10^7^ cells]	NU	0.7315	0.3646	0.6321	0.5228
	GRA	0.4468	0.1473	0.4388	0.3024
^99m^Tc-HDP labelling [MBq]	NU	3.184	0.463	3.417	0.820
	GRA	2.102	0.751	2.512	1.340
	NU-Co	0.626	0.171	0.725	0.332
	GRA-Co	0.635	0.197	0.714	0.365
^99m^Tc-HDP labelling per cells [MBq/10^4^ cells]	NU	0.0429	0.0134	0.0415	0.0101
	GRA	0.0345	0.0135	0.0293	0.0223
	NU-Co	0.0166	0.0302	0.0153	0.0174
	GRA-Co	0.0205	0.0222	0.0253	0.0164

^*99m*^*Tc-HDP*: ^*99m*^*Technetium-hydroxydiphosphonate.* NU: atrophic non-union derived MSC, GRA: graft (Reamer-Irrigator-Aspirator femur aspirate, Iliac crest) derived MSC, NU-Co: non-osteogenic differentiated corresponding control group for NU. GRA-Co: non-osteogenic differentiated corresponding control group for GRA. *SD* standard deviation, *IQR* inter quartile range
